# An observational study on diagnosis index of metabolic disease with blood-stasis

**DOI:** 10.1097/MD.0000000000021140

**Published:** 2020-07-02

**Authors:** Mi Mi Ko, Soobin Jang, Jeeyoun Jung

**Affiliations:** Clinical Medicine Division, Korea Institute of Oriental Medicine, Daejeon, Republic of Korea.

**Keywords:** blood stasis, herbal medicine, Korean medicine, metabolic disease, prospective observational study

## Abstract

**Introduction:**

Treating blood stasis is effective in treating obesity and metabolic diseases in traditional Korean medicine. The aim of this prospective observational study is to determine the effectiveness of the diagnosis index for metabolic diseases with blood stasis by analyzing clinical data and blood samples.

**Methods and analysis:**

We will perform a prospective observational study. Participants who meet the inclusion criteria will be recruited from the Dongguk university Ilsan Oriental hospital. The outcomes are resistin, serum amyloid P component, C-reactive protein, D-dimer, and blood stasis scores. In addition, the blood pressure, ankle–brachial pressure index, brachial–ankle pulse wave velocity, body mass index, waist circumference, and levels of blood lipid will be assessed.

**Discussion:**

Through this study, we could collect specific data for diagnosing metabolic diseases with blood stasis. Therefore, the findings of this study will provide a summary of the current state of evidence regarding the effectiveness of the diagnosis index in managing metabolic disease with blood stasis.

**Ethics and dissemination:**

The study was approved by the Institutional Review Board of the Dongguk University Ilsan Oriental Hospital (DUIOH-2018-09-001-007). The results will be published in a peer-reviewed journal and will be disseminated electronically and in print.

**Trial registration number:**

Clinical Research Information Service: KCT0003548

## Abstract 2

Supplemental Digital Content is available in the text

## 1 Introduction

Circulatory disturbance manifests as various symptoms caused by reduced blood flow from blood clotting or changes in blood composition. There are 2 types of circulatory disturbances: hemorrhage, meaning escape of blood from the blood vessels, and ischemia, meaning failure/restriction of blood flow to a particular part of the body.^[1]^ Probable conditions leading to circulatory disturbances include hypertension, cardiovascular diseases, diabetes, hyperlipidemia, and arteriosclerosis, which cause complications, such as angina, dementia, and paralysis.^[2–4]^

Typically, blood stasis refers to the condition in which blood in the body does not flow inside the blood vessels, which are obstructed, or in which body fluids are found in an area outside their original position. In oriental medicine, it is believed that blood stasis cuts off communication within the meridian pathways and causes diseases.^[5–8]^ Particularly, chronic and incurable diseases, such as pain, infertility, and cancer, and diseases caused by stress cannot be treated with modern medicine, and are associated with blood stasis.^[5,6]^ However, the current concept of blood stasis is unclear. Therefore, through studies on blood stasis, it is essential to establish a foundation for oriental medical treatment by identifying a highly scientific part from the traditional concept of blood stasis, defining this part scientifically and clearly, reinterpreting the relevant diseases from the modern viewpoint, and providing a standard for the diagnosis of chronic and incurable diseases.^[6,7]^

Blood stasis refers to all pathological conditions resulting in aggregation and modification of the red blood cells, which can cause hematological changes, such as an increase in blood viscosity and reduction in blood flow velocity.^[6,8,9]^ Therefore, all diseases caused by these conditions are referred to as blood stasis, and blood stasis can be modernly reinterpreted by scientifically clarifying the concepts of blood and blood vessels.

In Korea, the prevalence of metabolic diseases in people aged ≥30 years is 28.8% (ie, 1 out of 3 people). The average prevalence rate of metabolic diseases (including hypertension, diabetes, hypercholesterolemia, and cancer) has increased by 1.8%, from 16.1% in 2007 to 17.9% in 2013, and this trend is particularly high in individuals aged ≥60 years.^[10]^ Treating blood stasis is effective in treating obesity and metabolic diseases in traditional Korean medicine.^[8,11]^

This prospective observational study with an exploratory purpose is aimed at designing a Korean medicine diagnostic technique for metabolic diseases with blood stasis, particularly circulatory disturbances, to explore the related Korean medicine treatment options and evaluate their validity by analyzing clinical data.

## 2 Participants and methods

### 2.1 Study aims

This exploratory study is aimed at designing a Korean medicine diagnostic technique for metabolic diseases with blood stasis, particularly circulatory disturbances, to explore the related Korean medicine treatment options and evaluate their validity by analyzing clinical data and blood samples.

### 2.2 Study design/setting

**Figure 1:**
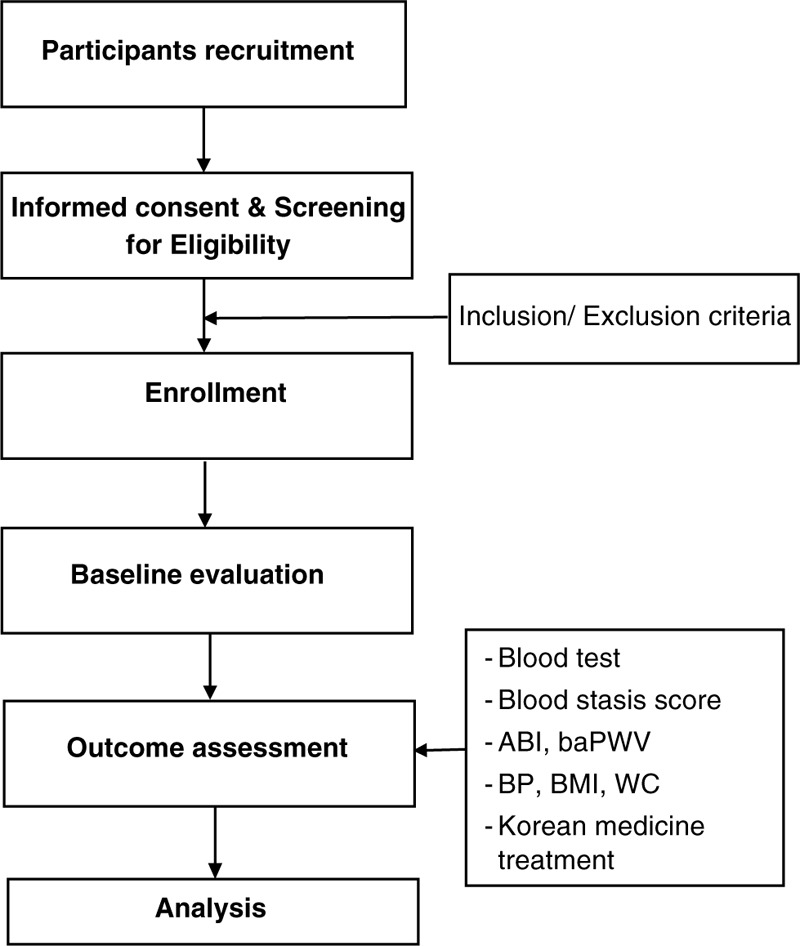
A flow chart of the study. ABI = ankle-brachial index, baPWV = brachial-ankle pulse wave velocity, BMI = body mass index, BP = blood pressure, Visit 1 = 7 days after screening, WC = waist circumstance.

This is a prospective observational study. We will recruit patients from the Dongguk University Ilsan Oriental Hospital (Ilsan) (Fig. 1). Table 1 shows the data collection and follow-up schedules.

**Table 1:**
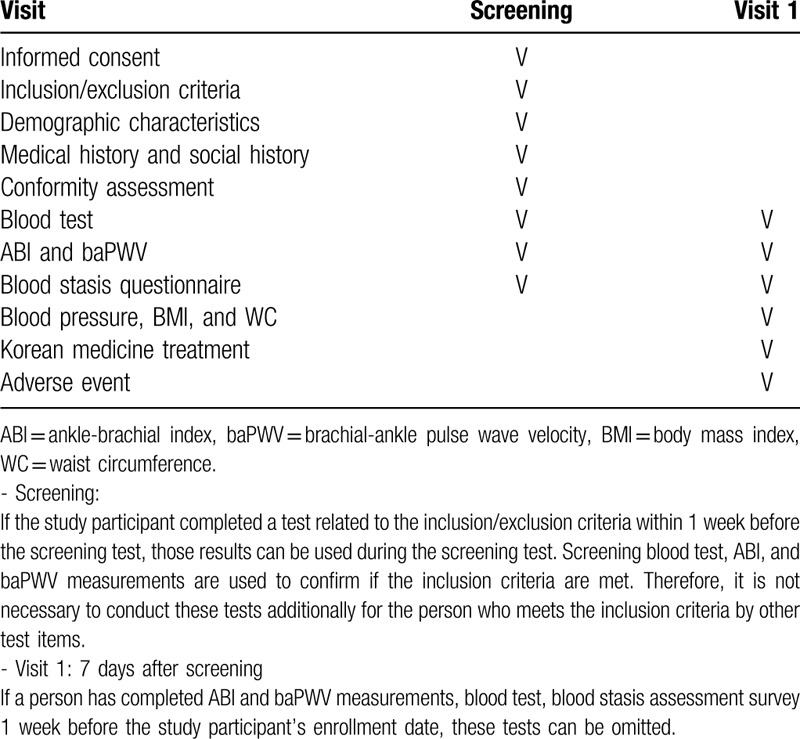
Schedule of study visits and assessments.

### 2.3 Study registration

This study is registered with the Clinical Research Information Service (https://cris.nih.go.kr/cris/en/): KCT0003548. Current protocol versions are 1.2.

### 2.4 Eligibility criteria

#### 2.4.1 Inclusion criteria

We will include patients who are eligible according to the following criteria:

1. Individuals aged between 20 and 70 years.
2. Individuals who voluntarily sign the informed consent form for the clinical study, or those who are incapable of providing voluntary agreement but whose legal representatives sign the informed consent form.
3. Individuals who are willing to cooperate and comply with regulations during the study period.
4. Individuals who consent for blood collection for the purpose of this study.

For the study participants who meet the aforementioned criteria, the selection criteria for each group are as follows:

1. Ankle–brachial index (ABI) <0.9 or triglycerides ≥150 mg/dL and high-density lipoprotein cholesterol <40 mg/dL in men and <50 mg/dL in women
2. Blood stasis score (≥9 points out of 32 total points)
  - Group I (circulatory disturbance I): Meets both aforementioned criteria (1 and 2).
  - Group II (circulatory disturbance II): Meets one of the aforementioned criteria (1 but not 2).
  - Group III (normal): Does not meet either criterion from the aforementioned criteria.

#### 2.4.2 Exclusion criteria

1. Individuals with mental illness or those unable to communicate
2. Critically ill patients who are unconscious and unable to communicate
3. Pregnant patients
4. Individuals currently on oriental medicine for the treatment of blood stasis
5. Individuals presenting with any other condition that could influence the assessment of this study, based on the investigator's discretion

### 2.5 Recruitment

The researcher will explain the aim of this study and the details of the procedures and will obtain informed consent from potential subjects prior to the collection of information. Participants will be free to withdraw at any time during the study, and this will not affect their clinical treatment. We encourage voluntary participation in this clinical study that uses human biological material, by posting recruitment posters on the walls of the entrance and elevators of the hospital and through newspapers and bus advertisements.

### 2.6 Intervention

Since this is an observational study, the treatment to improve circulatory disturbance is not restricted during the study. For a given period, treatment with Korean medicine, other Korean medical treatment, and prescription medication other than Korean medicine should be recorded. For Korean medicine, the name of the medicine, medicine composition, and treatment duration should be recorded.

For participants enrolled in the study after screening, we will perform blood tests; ABI and brachial–ankle pulse wave velocity measurement; blood stasis diagnosis questionnaire assessment; blood pressure, body mass index, and waist circumference measurement; and concomitant drug and Korean medical treatment assessment at visit 1 within 7 days of registration.

### 2.7 Outcomes measures

#### 2.7.1 Primary outcome

1. Resistin, serum amyloid P component, C-reactive protein, and D-dimer are the candidate indicators that seem effective in diagnosing blood stasis based on previous studies on blood stasis.^[12–15]^
2. Blood stasis score: A blood stasis questionnaire used to assess the presence/absence of blood stasis and to evaluate the blood stasis score was developed by the Korea Institute of Oriental Medicine through a blood stasis diagnosis technique development research project.^[16–18]^ It was executed for 5 years from 2013 to 2018, and it showed high reliability and validity.^[17,18]^ We extracted 31 questions related to metabolic disorders from the blood stasis questionnaire using the experts’ delphi method and validity analysis, in order to use it as an assessment tool in this study.^[19]^ By referring to the standard operating procedure of each question, a Korean medicine doctor will directly interview each patient and complete the blood stasis questionnaire (Supplemental Digital Content (Appendix 1)).

#### 2.7.2 Secondary outcome

1. ABI is an indicator used to evaluate the degree of lower extremity artery stenosis or occlusion.^[20]^ The lower value between the left and the right measurements will be recorded.
2. The brachial-ankle pulse wave velocity, an indicator of the brachial-ankle pulse wave velocity, is used to evaluate the presence or absence of the atherosclerosis progress in the main limb arteries.^[21]^
3. Total cholesterol, triglycerides, and high-density lipoprotein cholesterol values.
4. Blood pressure, body mass index, and waist circumference measurements.

### 2.8 Data collection and management

We will code the identification of patients. We will also enter data collected at each study visit into a paper case report form (CRF) with double data entry. Outcome data entered into the database will be verified against the source data on the paper CRFs. We will encourage patients to complete study and follow-up using text messages and personal calls.

### 2.9 Sample size calculation

This clinical study using human biological material is an exploratory study. Therefore, we have performed no effect size-based statistical calculation of the number of study participants. Considering the dropout rate, we aim to recruit 100 study participants from the Dongguk University Ilsan Oriental Hospital.

### 2.10 Statistical analysis

A basic analysis of the study results will compare the difference in evaluation indicators of groups I and II (with circulatory disturbances) with that of group III (without circulatory disturbances) at visit 1. All statistical analyses will be subjected to two-tailed tests, and the significance level will be set at 5%. A descriptive analysis of the evaluation items and blood indices for each group will be conducted to represent the categorical variables as n (%) and continuous variables as mean ± standard deviation. To compare the intergroup differences for the obtained outcome value for each item, categorical variables will be subjected to the chi-square test and Fisher exact test, whereas continuous variables will be analyzed using the *t* test and analysis of variance or nonparametric methods such as Mann-Whitney *U* test and Kruskal-Wallis test for those not meeting the normality criteria. Analysis of covariance will be performed when it is necessary to control underlying variables, such as demographic variables (sex, age, weight, and height). For data with a missing value, the last observation carried forward will be used through CRF inspection to replace the missing value.

### 2.11 Ethics and dissemination

The Institutional Review Board of the Dongguk University Ilsan Oriental Hospital (DUIOH-2018-09-001-007) approved the study. Written informed consent will be obtained from all study participants before enrollment in the study. The results will be published in a peer-reviewed journal and disseminated electronically and in print regardless of results.

## 3 Discussion

This prospective observational study is designed to investigate the effectiveness of the diagnosis index for metabolic diseases with blood stasis by analyzing clinical data. Treating blood stasis is effective in treating metabolic diseases in traditional Korean medicine,^[8,11]^ but there is insufficient evidence regarding the effectiveness of the diagnosis index in metabolic diseases with blood stasis. Findings of this study will provide a summary of the current state of evidence regarding the effectiveness of the diagnosis index in managing metabolic diseases with blood stasis. In addition, this study is expected to provide a basis for clinical trials to confirm the efficacy of Korean medicine for treatments of metabolic diseases with blood stasis.

## Author contributions

**Conceptualization:** Mi Mi Ko, Soobin Jang, Jeeyoun Jung

**Data curation:** Mi Mi Ko, Soobin Jang

**Methodology:** Mi Mi Ko, Soobin Jang, Jeeyoun Jung

**Resources:** Mi Mi Ko, Soobin Jang, Jeeyoun Jung

**Software:** Mi Mi Ko, Supervision: Jeeyoun Jung

**Writing – original draft:** Mi Mi Ko

**Writing – review & editing:** Mi Mi Ko, Soobin Jang, Jeeyoun Jung

## Supplementary Material

Supplemental Digital Content
