# Supplementary material for: An observational study on diagnosis index of metabolic disease with blood-stasis
Source: Medicine (Baltimore). 2020 Jul 2;99(27):e21140. doi: 10.1097/MD.0000000000021140 (PMC7337439; doi:10.1097/MD.0000000000021140)
Supplement: Supplemental Digital Content [file medi-99-e21140-s001.docx]

**Supplemental Digital Content**

**Appendix 1.** 15-item questionnaire for the diagnosis of blood stasis with metabolic diseases

| Question | | Response | | Score |
| --- | --- | --- | --- | --- |
| 1 | Angina pectoris | No | Yes | 3 points |
| 2 | Having chest pain without angina pectoris | No | Yes |  |
| 3 | Blackish red tongue | No | Yes |  |
| 4 | Ecchymosis of tongue | No | Yes |  |
| 5 | Stabbing pain | No | Yes |  |
| 6 | Sublingual varicosities | No | Yes | 2 points |
| 7 | Dark purple of palate mucosa | No | Yes |  |
| 8 | Blackish red lips | No | Yes |  |
| 9 | Blackish red gingiva | No | Yes |  |
| 10 | Chronic pain in joint/palsies and numbness | No | Yes |  |
| 11 | Pain at night | No | Yes |  |
| 12 | Bruised easily | No | Yes |  |
| 13 | Dark colouration of periocular region | No | Yes | 1 point |
| 14 | A dark colouration of the face | No | Yes |  |
| 15 | Scaly and rough skin | No | Yes |  |
